# Association between tea consumption and colorectal cancer: a systematic review and meta-analysis of a population-based study

**DOI:** 10.1186/s12876-023-02928-8

**Published:** 2023-08-31

**Authors:** Yu Huang, Qiang Chen, Yating Liu, Ruoxi Tian, Xu Yin, Yaoguang Hao, Yang Yang, Jian Yang, Zongxuan Li, Suyang Yu, Hongyan Li, Guiying Wang

**Affiliations:** 1 Department of Gastrointestinal Surgery, The Third Hospital of Hebei Medical University, Shijiazhuang, 050051 P.R. China; 2 Department of Clinical Medical College, Tianjin Medical University, Tianjin, P.R. China; 3 Department of Thoracic Surgery Gastrointestinal Surgery, the Third Hospital of Hebei Medical University, Shijiazhuang, P.R. China; 4 Department of Vascular Surgery Gastrointestinal Surgery, the Third Hospital of Hebei Medical University, Shijiazhuang, P.R. China

**Keywords:** Tea consumption, CRC, Population-based study, Meta-analysis, Systematic review

## Abstract

**Purpose:**

A meta-analysis study was performed to systematically assess the association between tea consumption and CRC risk.

**Methods:**

Cochrane Library, Embase, PubMed, and Web of Science were retrieved to collect articles in English since 24 July 2023. Databases were searched and evaluated by two reviewers independently.We screened the literature based on inclusion and exclusion criteria. After determining the random effect model or fixed utility model based on a heterogeneity test, odds ratios (ORs) and 95% confidence intervals (CIs) were calculated.

**Results:**

We included fourteen articles in this meta-analysis. We analyzed the data using a random effect model to explore the association between tea consumption and CRC because of apparent heterogeneity (*P* < 0.001, I2 = 99.5%). The combined results of all tests showed that there is no statistically significant association between tea consumption and CRC risk (OR = 0.756, 95%CI = 0.470–1.215, *P* = 0.247). Subsequently, subgroup analysis and sensitivity analysis were performed. Excluding any single study, the overall results ranged from 0.73 (95%CI = 0.44–1.20) to 0.86 (95%CI = 0.53–1.40). It was determined that there was no significant publication bias between tea consumption and CRC risk (*P* = 0.064) by Egger's tests.

**Conclusions:**

The results indicated that tea consumption may not be significantly associated with the development of CRC.

**Implications of key findings:**

Tea reduces colon cancer risk by 24%, but the estimate is uncertain. The actual effect on risk can range from a reduction of 51% to an increase of 18%, but regional and population differences may cause differences.

## Introduction

Colorectal cancer (CRC) is one of the most common malignant tumors of the digestive system [1]. CRC has been lately reported as the third most common cancer in the world, but it is the second most common cause of death [2]. Despite noticeable improvement being achieved in the survival of CRC patients through improvements in surgical, oncological treatment, planning, and follow-up, its global incidence has been increasing in recent years [3]. Many therapeutic options against CRC have been developed, but the five-year survival rate of patients with metastatic CRC is dismal as low as 12% as compared to 64% for CRC in general [1]. CRC has become an increasingly serious global health issue and it is essential to prevent the occurrence of CRC as early as possible.

Studies around the world have shown that risk factors for CRC including inflammatory bowel diseases, first-degree relatives with CRC, obesity, lack of physical activity and obesity, smoking, red meat consumption, and low intake of fruits and vegetables [4]. Dietary factors play a key role in CRC carcinogenesis, according to epidemiological studies [5], which has been considered an important strategy for CRC prevention [6].

Tea is a favorable beverage throughout the world, and is recognized as a chemical preventive agent for some diseases. Many studies have been carried out to explore the association between tea consumption and CRC, and evidences have shown that tea may contain some protective ingredients against CRC [7–9]. It has been found that, tea polyphenols, one of the most abundant components in tea, can inhibit tumor development by promoting tumor cells apoptosis, inhibiting proliferation and angiogenesis via regulating some signaling pathways such as Ras-MAPK [10]. Despite this, the findings remain controversial. The results of some studies have also indicated that consumption of tea is not associated with a decreased risk of CRC [11, 12].

Over the past few decades, numerous studies have evaluated the association between tea consumption and CRC morbidity and mortality, however the conclusion is inconsistent and no consensus has been reached. Therefore, in the present study, we conducted a population-based meta-analysis to assess the association between tea consumption and CRC.

## Materials and methods

### Literature retrieval

Four major literature databases (Cochrane Library, Embase, PubMed, and Web of Science) were searched to retrieve English articles published since 24 July 2023. Retrieval imposed restrictions on the document types. Detailed information on each was provided below. The terms "Colorectal cancer" and "Tea" were used as subject terms. "Colorectal Neoplasm", “Black Tea”, “Black Teas”, “Green Tea”, “Green Teas”, and “Tea consumption” etc. were used as free words. To improve the retrieval results, we combined the subject word with a free word. Reference lists from the identified publications were reviewed to identify additional research articles. To prevent research from being missed, the references in the studies retrieved from the online databases and previously published systematic reviews were also manually searched to further identify relevant studies. Detailed literature retrieval processes in the PubMed database were listed in Table 1.

**Table 1:** PubMed database literature search format

**Search number**	**Query**
#1	"Colorectal Neoplasms"[MeSH Terms]
#2	"colorectal neoplasms"[Title/Abstract] OR "colorectal neoplasm"[Title/Abstract] OR "neoplasm colorectal"[Title/Abstract] OR "neoplasms colorectal"[Title/Abstract] OR "colorectal tumors"[Title/Abstract] OR "colorectal tumor"[Title/Abstract] OR "tumor colorectal"[Title/Abstract] OR "tumors colorectal"[Title/Abstract] OR "colorectal cancer"[Title/Abstract] OR "cancer colorectal"[Title/Abstract] OR "cancers colorectal"[Title/Abstract] OR "colorectal cancers"[Title/Abstract] OR "colorectal carcinoma"[Title/Abstract] OR "carcinoma colorectal"[Title/Abstract] OR "carcinomas colorectal"[Title/Abstract] OR "colorectal carcinomas"[Title/Abstract]
#3	#1 OR #2
#4	"Tea"[MeSH Terms]
#5	"Tea"[Title/Abstract] OR "black tea"[Title/Abstract] OR "black teas"[Title/Abstract] OR "tea black"[Title/Abstract] OR "teas black"[Title/Abstract] OR "green tea"[Title/Abstract] OR "green teas"[Title/Abstract] OR "tea green"[Title/Abstract]OR "teas green"[Title/Abstract] OR "tea consumption"[Title/Abstract]
#6	#4 OR #5
#7	"Prospective Studies"[MeSH Terms]
#8	"prospective studies"[Title/Abstract] OR "prospective study"[Title/Abstract] OR "studies prospective"[Title/Abstract] OR "study prospective"[Title/Abstract]
#9	#7 OR #8
#10	#3 AND #6 AND #9

### Study selection

Literatures were selected for meta-analysis when met the following criteria: (1) The association between tea consumption and colorectal cancer was examined in a cohort of individuals who were in good health, and the diagnosis of colorectal cancer was determined based on the criteria outlined in the AJCC 8th edition diagnostic guidelines for this particular type of cancer; (2) case–control studies, cohort studies, and a randomized controlled trial; (3) assessed the association between tea consumption and CRC risk; (4) provided the number of participants; and (5) The risk estimates were reported with corresponding 95% confidence intervals (95% CIs). Meanwhile, studies were excluded with one or more of the following criteria: (1) review articles; (2) animal trials; (3) conference papers; (4) data unavailable to be extracted; and (5) inaccessible full text through various approaches on tea consumption.

### Data extraction and quality assessment

The full text of all articles included was reviewed. Data abstraction and full-text screened review were carried out independently by two authors, and discrepancies were corrected by the third. To remove duplicates, we imported the extracted study into the Endnote Software X9.0, followed by the screening of titles and abstracts by two authors. PRISMA statement guidelines were followed throughout all processes [13]. Data collection was conducted using standardized forms developed by the research team. Data extraction included the following information: study characteristics, such as study types, authors, year of publication, number of patients and sample size. An analysis of clinical indicators and data was conducted: (1) case/participants; (2) population of country; (3) tea types; (4) tea consumption (< 1 cup vs. ≥ 1 cup); (5) Cancer sites; and (6) age. The study quality assessment was performed following the Newcastle–Ottawa Scale [14]. The scoring system assessed three aspects of a study: selections (representativeness of cohort and exposure assessment); comparability (confounding determination) and outcomes (assessment of the outcome and follow-up). The studies were rated based on selection, comparability, exposure and outcome, and scored with a maximum of nine points. There were two categories of papers: high-quality (study score ≥ *7) and low-quality (study score < *7) [15, 16]. We rated them based on the following: Is the case definition adequate?,Representativeness of the cases, Definition of Controls, Comparability of cases and controls on the basis of the design or analysis, Ascertainment of exposure, Same method of ascertainment for cases and controls, Non response.

### Statistical analysis

The meta-analyses were performed using Stata17.0. Since the indices collected in this study were dichotomous variables, the odds ratio (OR) was used as the effect size in the statistical analysis. The I2 index and Cochran's Q tests were employed to quantify incoherence and heterogeneity between studies, respectively. I2 was evaluated as a measure of heterogeneity across studies, which was interpreted as not significant (0%-40%), moderate heterogeneity (30%-60%), substantial heterogeneity (50%-90%), or large heterogeneity (75%-100%) [17]. If there was significant heterogeneity between studies, a random effect model was used; otherwise, a fixed effect model was used. A sensitivity and subgroup analysis was performed to explore potential causes of heterogeneity. There were several confounding factors, including geographic location, tea types, cancer sites, quality scores, and study types. We assessed the sources of heterogeneity by analyzing the previously described factors in the subgroups. Meanwhile, analyses of sensitivity were conducted to evaluate the robustness of the main outcomes. Furthermore, Egger’s correlation tests regressed the publication bias, the *P* value at 0.05 (*) was considered statistically significant, and the test results were attached to the paper [18].

## Results

### Search results

**Fig. 1:**
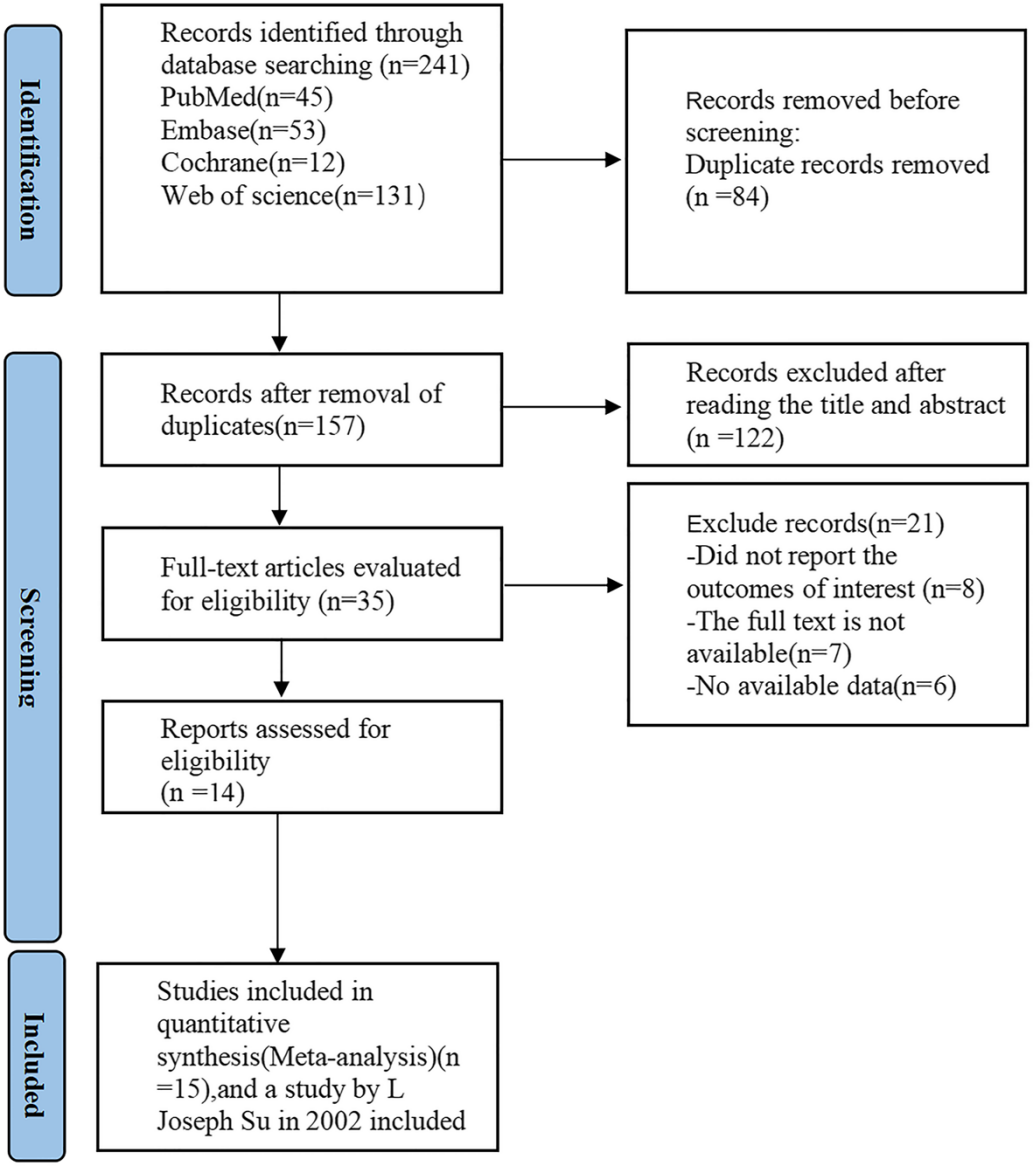
PRISMA flow diagram of the study process. PRISMA, Preferred Reporting Items for Systematic review and Meta-analysis

A total of 239 relevant studies were retrieved from the initial literature review. Duplicate articles were firstly removed among predefined databases based solely on titles. Additionally to 84 duplicate articles, the remaining 155 studies were also screened by reviewing titles and abstracts. In addition, 120 studies based on animals, review articles, and case reports were also ruled out from this work. A comprehensive review of 35 studies was conducted. 8 articles were excluded due to missing results of interest, 7 articles were ruled out because of inaccessible full texts, and 6 with data unavailable. Ultimately 14 articles were included in the meta-analysis [8, 19–31]. Among them, L Joseph Su’s study in 2002 included two cohort studies, so a total of 15 studies were included for quantitative synthesis (meta-analysis). The process of literature retrieval was shown in Fig. 1.

### Study characteristics, and quality assessment

A summary of the characteristics of the included studies were presented in Table 2. The included 15 studies were published between 1986 and 2015, which included total 2,693,030 participants. These studies were conducted in Asia (4 in China, 1 in Singapore), North America (4 in the United States), Europe (2 in the United Kingdom, 2 in France, 1 in Italy), and Oceania (1 in Australia). In addition, 3 were case–control studies, 11 were cohort studies, and 1 was a randomized controlled trial. Tea consumption showed an inherent relationship with CRC risk in all studies.

**Table 2:** Characteristics of included studies

**Author**	**Year**	**study type**	**Events**	**Country**	**Age**	**NOS**	**Tumor site**	**Types of Tea**
James R	2015	Case–control study	3672	America	40–85	5	colon, rectum	Any tea
Vincent K. Dik	2013	Prospective cohort study	477,071	French	64.7 ± 8.3	7	colon, rectum	Any tea
C Dominianni	2013	Prospective cohort study	53,542	UK	NA	7	colon, rectum	Green tea
Chadwick John Green	2014	Case–control study	1802	Australia	40–79	4	colon, rectum	Any tea
Terryl J. Hartman	2015	Randomized controlled trials	27,108	French	50–69	6	colon, rectum	Any tea
L.K. Heilbrun	1986	Prospective cohort study	7938	UK	45–68	5	colon, rectum	Black tea
Xinyi L	2019	Prospective cohort study	455,981	Chinese	30–79	7	colon, rectum	Any tea
Sarah Nechuta	2012	Prospective cohort study	68,522	Chinese	40–70	7	colon, rectum	Green tea
Rashmi Sinha	2012	Prospective cohort study	343,975	America	50–71	8	colon, rectum	Any tea
L Joseph Su cohort1	2002	Prospective cohort study	12,335	America	25–74	5	colon	Green tea
L Joseph Su cohort2	2002	Prospective cohort study	12,335	America	25–74	5	colon	Green tea
Can-Lan Sun	2007	Prospective cohort study	546,563	Singapore	45–74	6	colon, rectum	Green tea, Black tea
Alessandra Tavana	1997	Case–control study	10,569	Italy	19–79	4	colon, rectum	Any tea
Gong Yang	2007	Prospective cohort study	397,841	Chinese	40–70	7	colon, rectum	Green tea
Gong Yang	2011	Prospective cohort study	273,776	Chinese	40–74	7	colon, rectum	Green tea

As shown in Tables 2 and 3, the NOS scores for all included studies ranged from 4 to 8 points. Seven in fifteen studies were considered to be high quality. In terms of selection and outcome bias, all studies conformed to the inclusion criteria.

**Table 3:** Quality assessment for observation studies by Newcastle Ottawa Scale

Study	Is the case definition adequate?	Representativeness of the cases	Definition of Controls	Comparability of cases and controls on the basis of the design or analysis	Ascertainment of exposure	Same method of ascertainment for cases and controls	Non response	Total scores
James R 2015	*	-	-	*	*	*	*	5
Vincent K. Dik 2013	*	*	*	*	*	*	*	7
C Dominianni 2013	*	*	*	*	*	*	*	7
Chadwick John Green 2014	*	-	-	*	*	*	-	4
Terryl J. Hartman 2015	*	*	*	*	*	*	-	6
L.K. Heilbrun 1986	*	-	-	*	*	*	*	5
Xinyi L 2019	*	*	*	*	*	*	*	7
Sarah Nechuta 2012	*	*	*	*	*	*	*	7
Rashmi Sinha 2012	*	*	*	**	*	*	*	8
L Joseph Su cohort1 2002	*	-	-	*	*	*	*	5
L Joseph Su cohort2 2002	*	-	-	*	*	*	*	5
Can-Lan Sun 2007	*	*	*	*	*	*	-	6
Alessandra Tavana 1997	*	-	-	*	*	*	-	4
Gong Yang 2007	*	*	*	*	*	*	*	7
Gong Yang 2011	*	*	*	*	*	*	*	7

### Tea consumption and CRC risk

**Fig. 2:**
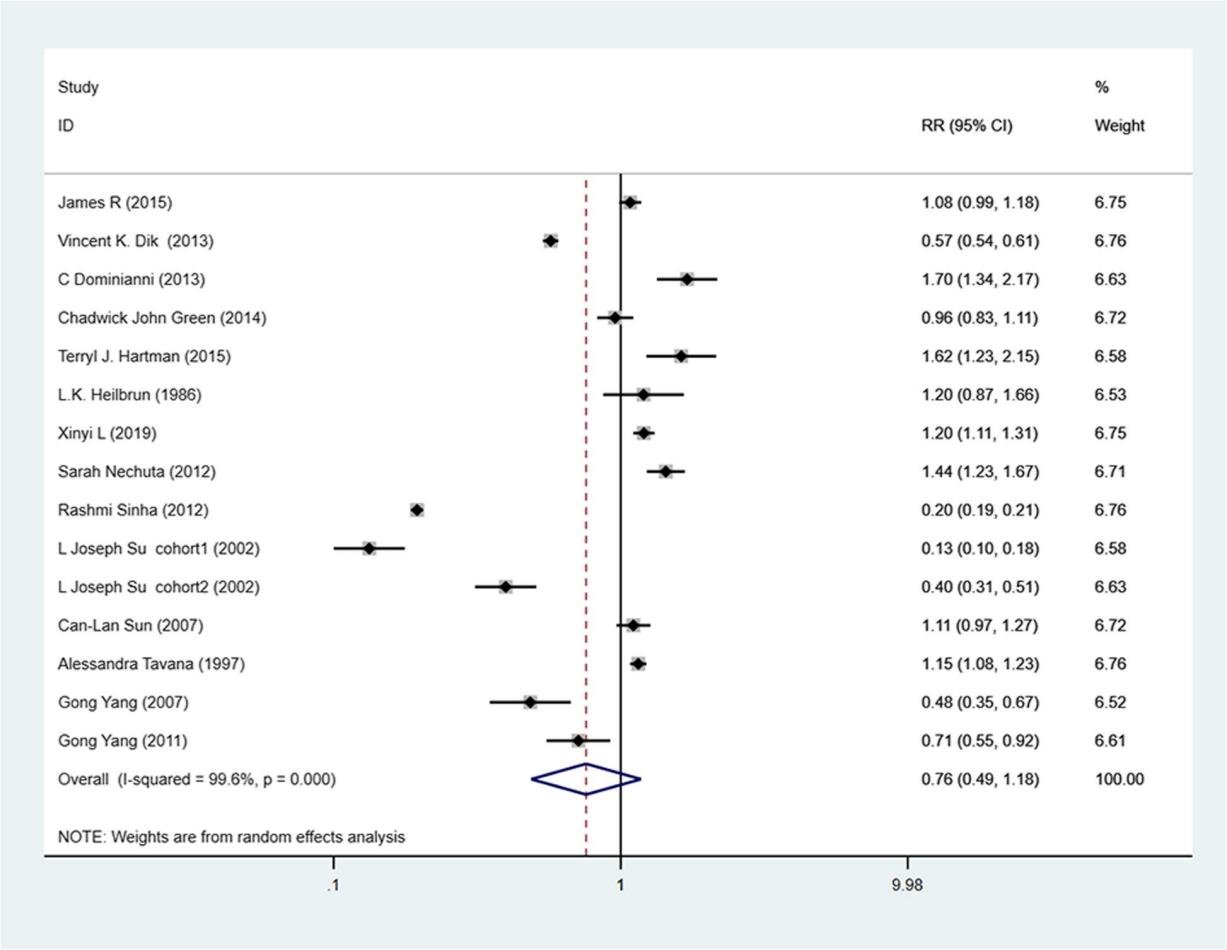
Forest plot of tea consumption and colorectal cancer risk

In Fig. 2, RRs from 15 studies were extracted after multivariable correction. A random effect model was used for data analysis to evaluate the association between tea consumption and CRC because of apparent heterogeneity (*P* < 0.001, I^2^ = 99.6%). The results of the combined test were RR = 0.758,95%CI 0.489–1.176, *P* = 0.216. Based on the combined results of all tests, no statistically significant association could be found between tea consumption and CRC risk (RR = 0.758, 95%CI 0.489–1.176, *P* = 0.216). Considering that the study results indicated the existence of significant heterogeneity, subgroup analysis, and sensitivity analysis were conducted in the subsequent study to explore the source of heterogeneity.

### Subgroup analysis

Subsequently, we categorized the studies by geographic regions (Subgroups were set up according to countries of the recruited population), amount of tea consumption, types of tea, sites of cancer, quality scores, types of study, and year of publication (Table 4). Except for geographic regions and types of tea, no statistically significant differences were found in other subgroups. The results indicated that neither group was a potential source of heterogeneity in meta-analyses. In subgroup stratified by geographic regions (countries of the study population), data from American subgroup analysis suggested that tea consumption might protect against CRC (RR = 0.326, 95%CI 0.110–0.908). Conversely, data from the UK (RR = 1.454, 95%CI 1.031–2.050) and Italian subgroup (RR = 1.151, 95%CI 0.079–1.229, *P* < 0.001) exhibited opposite results. Therefore, tea consumption might be associated with CRC to some degree. It is worth noting that, in subgroups of tea types, green tea consumption was associated with the reduced risk of CRC (RR = 0.049, 95%CI 0.031–0.067, *P* < 0.001).

**Table 4:** Subgroup analyses of tea consumption and colorectal cancer risk

**Group**	**Studies (n)**	**RR (95% CI)**	***P***	**Heterogeneity test**	
				***P***	***I2* (%)**
Total	15	0.758 (0.489–1.176)	0.216	0	99.6
Design					
Case–control	3	1.079 (0.985–1.182)	0.102	0.061	64.2
Cohort	11	0.645 (0.371–1.122)	0.120	0	99.6
Randomized controlled trials	1	1.625 (1.228–2.150)	0.001	NA	NA
Tea source					
Green tea	9	0.049 (0.031–0.067)	0.001	0	98.6
Black tea	2	0.020 (-0.018–0.058)	0.295	0	97.5
Any tea	13	0.012 (0.009–0.014)	0	0	99.8
Amount of tea					
< 1	12	0.008 (0.006–0.009)	0	0	99.6
≥ 1	13	0.012 (0.010–0.014	0	0	99.8
Area1					
America	4	0.326 (0.110–0.908)	0.043	0	99.7
France	2	0.954 (0.342–0.660)	0.928	0	98
UK	2	1.454 (1.031–2.050)	0.033	0.087	65.9
Australia	1	0.957 (0.828–1.106)	0.553	NA	NA
China	4	0.906 (0.635–1.292)	0.585	0	94
Singapore	1	1.107 (0.966–1.269)	0.142	NA	NA
Italy	1	1.151 (1.079–1.229)	0	NA	NA
Location					
Colon	11	0.607 (0.365–2.264)	0.837	0	99.7
Rectal	9	1.159 (0.454–2.960)	0.758	0	99.4
Publication year					
< 2010	6	0.587 (0.337–1.023)	0.060	0	98.3
≥ 2010	9	0.899 (0.493–1.639)	0.728	0	99.7
Quality score					
< 7	8	0.788 (0.573–1.082)	0.141	0	97.6
≥ 7	7	0.732 (0.360–1.488)	0.389	0	99.7

### Sensitivity analysis

**Fig. 3:**
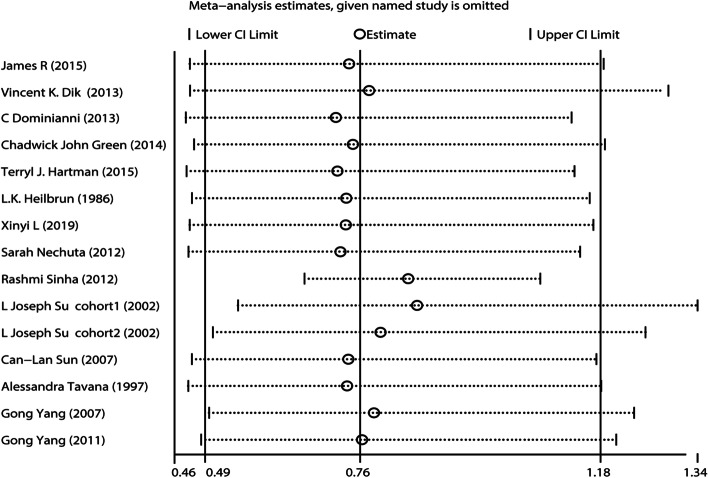
Sensitivity analysis results

Potential sources of heterogeneity were investigated using a sensitivity analysis. The results of the sensitivity analysis were shown in Fig. 3. Excluding any single study, the overall results ranged from 0.49 (95%CI = 0.46–0.66) to 1.18 (95%CI = 1.07–1.34), implying that the main results were robust.

### Publication bias

**Fig. 4:**
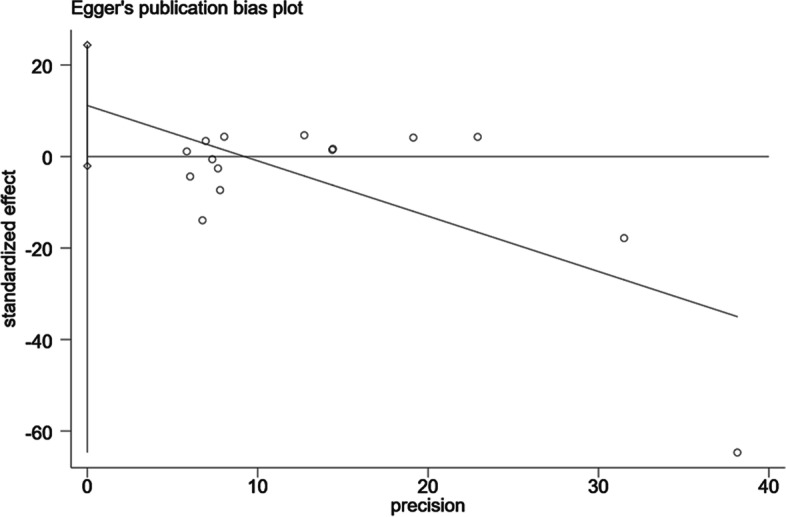
Results of publication bias

To detect publication bias in the included studies, Egger tests were conducted and the findings were visualized as well (Fig. 4). Data showed that there was no significant publication bias between tea consumption and CRC risk (*P* = 0.064) by Egger’s tests.

## Discussion

CRC has emerged as a challenge threatening individual health globally [2]. This poses a serious threat to human health. It has been found previously that, tea extracts might perform some anti-cancer effects [9, 24, 32]. However, conclusions from the studies about the relationship between tea consumption and CRC in vivo and in vitro remain controversial [33, 34]. Studies conducted in Japan has found no association between tea consumption and CRC [35, 36]. While in a study performed by Yang G et al. in China has indicated that tea consumption may reduce the risk of CRC [31].

In the present study, we assessed the association between tea consumption and CRC risk comprehensively using a meta-analysis, and found that from the data so far, from whole tea consumption did not significantly reduce the risk of CRC. In addition,according to subgroup analysis,the dose–response relationship did not demonstrate a significant inversion between daily tea consumption and CRC risk. According to Zhang et al., higher tea consumption was correlated with a reduced risk of CRC [37]. Similar results have also been found by Wang et al. and Chen et al. have shown that tea intake can reduce the risk of CRC development [7, 38]. However, Yu et al. found no apparent relationship between the two aspects [39, 40]. Nevertheless, evidences from Zhu et al. showed that tea drinking may protect women against CRC [41]. In addition, they excluded case–control studies, which have a selection bias that limits their interpretation. In a meta published in 2020, one comprehensive report that summarizes the internal relationship between tea drinking and the development of cancer whereas it fails to elucidate the role of tea drinking in the occurrence and development of CRC [42].

Also in this study, the opposite relationship between tea consumption and CRC risk was found in the tea drinkers from America, the UK, and Italy. Possible affecting factors might be regional differences, individual differences, and the response to tea consumption by a person. Among the green tea drinkers, tea consumption was correlated with the lower risk of CRC, which was considered to be a protective factor. The possible cause could be the anti-cancer properties of tea polyphenols in green tea. Colorectal cancer might be modified through a variety of intracellular and extracellular processes, such as antioxidant activity, inflammation reduction, gut microbiota alterations, enzymatic inhibition in lipid or glucose metabolism and epigenetic changes [43–46]. In animal studies, green tea extract has been shown to prevent the occurrence and formation of precancerous lesions in the colon [47, 48].

This analysis recruited 2.7 million participants, approximately 700,000 more than previous projects. In this study, all relevant prospective studies (*n* = 15) with a large number of participants and abundant data were included, providing a higher degree of statistical power. There are still some limitations in this study: Firstly, the current meta-analyses failed to eliminate heterogeneity, either in the population or subgroup analyses. Secondly, although gender, age, and smoking are confounding factors in most studies, other potentially important variables, such as alcohol and fruit, have been neglected. The third point is that CRC is extremely complex and heterogeneous, with significant differences in incidence and etiology. This heterogeneity cannot be eliminated in the current model. Fourthly, because some included literature has a relatively small sample size, which may have limited statistical power, making it difficult to generalize the results. Last but not least, previous meta-analyses have used different ways to assess tea consumption. Even though some tea consumption was converted to cups per day prior to analysis, certain measurement errors were made in the original estimation of tea consumption.

It is well known that genetic and environmental factors are primarily responsible for the development of CRC. In recent years, CRC incidence has increased worldwide due to the advancement of diagnosis methods, technology, and improved health awareness among the public. Unfortunately, with the development of society, people’s dietary habits and lifestyles are also changing, resulting in the continuous breeding of CRC and other diseases. Tea has gained increasing attention for its effects on human health as one of the most popular beverages in the world. Numerous studies, including animal and cell experiments [49], have reported that tea extracts have anticancer effects on cancer development and progression. From a cellular and molecular perspective, the specific mechanism of CRC induced by tea consumption remains unclear and needs to be fully investigated. In designing future prospective studies, the experiences summarized in this article can be taken into account and stricter measurement criteria can be established to ensure the accuracy and reliability of the results as well as to minimize the interference of confounding factors.

However, this paper exhibits certain limitations that warrant further investigation. First of all, the high heterogeneity was observed in the findings of this study. Subsequently, we conducted subgroup analysis, sensitivity analysis, and assessed publication bias in order to identify the underlying cause of this heterogeneity. However, our investigation did not reveal any articles exhibiting substantial bias. The high level of heterogeneity observed in the original studies investigating the association between tea consumption and colorectal cancer risk can be attributed to several factors that are challenging to reconcile. These factors encompass the diverse characteristics of the patients involved, such as their geographical locations, racial backgrounds, and age distributions. Additionally, variations in the stages of colorectal cancer, the quantity and varieties of tea consumed, and the design and quality of the studies themselves contribute to this heterogeneity. Despite these inherent differences, the present study retains the ability to partially elucidate the underlying connection between tea consumption and the initiation and progression of colorectal cancer. Additionally, possible publication bias: While Egger’s test suggested no significant publication bias, it is important to consider that negative or non-significant results might be less likely to be published, leading to potential publication bias. This limitation should be acknowledged and discussed.

Taken together, this meta-analysis suggests that tea consumption may not be linked to the development of CRC. These relationships still need to be confirmed by additional well-designed large prospective studies and randomized clinical trials.

## Conclusion

Based on the studied samples of patients, the meta-analysis shows that tea reduces colon cancer risk by 24%, but the estimate is uncertain. The actual effect on risk can range from a reduction of 51% to an increase of 18%, but regional and population differences may cause differences.

## Data Availability

The data that support the findings of this study are available from the corresponding author upon reasonable request.

## References

[1] Siegel RL, Miller KD, Fuchs HE, Jemal A (2022). Cancer statistics, 2022. CA Cancer J Clin.

[2] Sung H, Ferlay J, Siegel RL, Laversanne M, Soerjomataram I, Jemal A, Bray F (2021). Global Cancer Statistics 2020: GLOBOCAN Estimates of Incidence and Mortality Worldwide for 36 Cancers in 185 Countries. CA Cancer J Clin.

[3] Brody H (2015). Colorectal cancer. Nature.

[4] Johnson CM, Wei C, Ensor JE, Smolenski DJ, Amos CI, Levin B, Berry DA (2013). Meta-analyses of colorectal cancer risk factors. Cancer Causes Control.

[5] Song M, Garrett WS, Chan AT (2015). Nutrients, foods, and colorectal cancer prevention. Gastroenterology.

[6] Arnold M, Sierra MS, Laversanne M, Soerjomataram I, Jemal A, Bray F (2017). Global patterns and trends in colorectal cancer incidence and mortality. Gut.

[7] Chen Y, Wu Y, Du M, Chu H, Zhu L, Tong N, Zhang Z, Wang M, Gu D, Chen J (2017). An inverse association between tea consumption and colorectal cancer risk. Oncotarget.

[8] Green CJ, de Dauwe P, Boyle T, Tabatabaei SM, Fritschi L, Heyworth JS (2014). Tea, coffee, and milk consumption and colorectal cancer risk. J Epidemiol.

[9] Luo KW, Xia J, Cheng BH, Gao HC, Fu LW, Luo XL (2021). Tea polyphenol EGCG inhibited colorectal-cancer-cell proliferation and migration via downregulation of STAT3. Gastroenterol Rep (Oxf).

[10] Jin H, Tan X, Liu X, Ding Y (2010). The study of effect of tea polyphenols on microsatellite instability colorectal cancer and its molecular mechanism. Int J Colorectal Dis.

[11] Michels KB, Willett WC, Fuchs CS, Giovannucci E (2005). Coffee, tea, and caffeine consumption and incidence of colon and rectal cancer. J Natl Cancer Inst.

[12] Suzuki Y, Tsubono Y, Nakaya N, Koizumi Y, Suzuki Y, Shibuya D, Tsuji I (2005). Green tea and the risk of colorectal cancer: pooled analysis of two prospective studies in Japan. J Epidemiol.

[13] Liberati A, Altman DG, Tetzlaff J, Mulrow C, Gøtzsche PC, Ioannidis JP, Clarke M, Devereaux PJ, Kleijnen J, Moher D (2009). The PRISMA statement for reporting systematic reviews and meta-analyses of studies that evaluate healthcare interventions: explanation and elaboration. BMJ (Clinical research ed).

[14] Stang A (2010). Critical evaluation of the Newcastle-Ottawa scale for the assessment of the quality of nonrandomized studies in meta-analyses. Eur J Epidemiol.

[15] Banke-Thomas AO, Madaj B, Charles A, van den Broek N (2015). Social Return on Investment (SROI) methodology to account for value for money of public health interventions: a systematic review. BMC Public Health.

[16] Gosselin V, Boccanfuso D, Laberge S (2020). Social return on investment (SROI) method to evaluate physical activity and sport interventions: a systematic review. Int J Behav Nutr Phys Act.

[17] Winkley K, Upsher R, Stahl D, Pollard D, Kasera A, Brennan A, Heller S, Ismail K (2020). Psychological interventions to improve self-management of type 1 and type 2 diabetes: a systematic review. Health Technol Assess.

[18] Egger M, Smith GD, Phillips AN (1997). Meta-analysis: principles and procedures. BMJ.

[19] Cerhan JR, Putnam SD, Bianchi GD, Parker AS, Lynch CF, Cantor KP (2001). Tea consumption and risk of cancer of the colon and rectum. Nutr Cancer.

[20] Dik VK, Bueno-de-Mesquita HB, Van Oijen MG, Siersema PD, Uiterwaal CS, Van Gils CH, Van Duijnhoven FJ, Cauchi S, Yengo L, Froguel P (2014). Coffee and tea consumption, genotype-based CYP1A2 and NAT2 activity and colorectal cancer risk-results from the EPIC cohort study. Int J Cancer.

[21] Dominianni C, Huang WY, Berndt S, Hayes RB, Ahn J (2013). Prospective study of the relationship between coffee and tea with colorectal cancer risk: the PLCO Cancer Screening Trial. Br J Cancer.

[22] Hartman TJ, Tangrea JA, Pietinen P, Malila N, Virtanen M, Taylor PR, Albanes D (1998). Tea and coffee consumption and risk of colon and rectal cancer in middle-aged Finnish men. Nutr Cancer.

[23] Heilbrun LK, Nomura A, Stemmermann GN (1986). Black tea consumption and cancer risk: a prospective study. Br J Cancer.

[24] Li X, Yu C, Guo Y, Bian Z, Shen Z, Yang L, Chen Y, Wei Y, Zhang H, Qiu Z (2019). Association between tea consumption and risk of cancer: a prospective cohort study of 0.5 million Chinese adults. Eur J Epidemiol.

[25] Nechuta S, Shu XO, Li HL, Yang G, Ji BT, Xiang YB, Cai H, Chow WH, Gao YT, Zheng W. Prospective cohort study of tea consumption and risk of digestive system cancers: results from the Shanghai Women’s Health Study. Am J Clin Nutr. 2012;96(5):1056–63. 10.3945/ajcn.111.031419 PMC3471195 23053557

[26] Sinha R, Cross AJ, Daniel CR, Graubard BI, Wu JW, Hollenbeck AR, Gunter MJ, Park Y, Freedman ND (2012). Caffeinated and decaffeinated coffee and tea intakes and risk of colorectal cancer in a large prospective study. Am J Clin Nutr.

[27] Su LJ, Arab L (2002). Tea consumption and the reduced risk of colon cancer – results from a national prospective cohort study. Public Health Nutr.

[28] Sun CL, Yuan JM, Koh WP, Lee HP, Yu MC (2007). Green tea and black tea consumption in relation to colorectal cancer risk: the Singapore Chinese Health Study. Carcinogenesis.

[29] Tavani A, Pregnolato A, La Vecchia C, Negri E, Talamini R, Franceschi S (1997). Coffee and tea intake and risk of cancers of the colon and rectum: a study of 3,530 cases and 7,057 controls. Int J Cancer.

[30] Yang G, Shu XO, Li H, Chow WH, Ji BT, Zhang X, Gao YT, Zheng W (2007). Prospective cohort study of green tea consumption and colorectal cancer risk in women. Cancer Epidemiol Biomarkers Prev.

[31] Yang G, Zheng W, Xiang YB, Gao J, Li HL, Zhang X, Gao YT, Shu XO. Green tea consumption and colorectal cancer risk: a report from the Shanghai Men’s Health Study. Carcinogenesis. 2011;32(11):1684–8. 10.1093/carcin/bgr186 PMC3246881 21856996

[32] Thomas R, Greef B, McConnachie A, Stanley B, Williams M. Dietary consumption of tea and the risk of prostate cancer in the Prostate, Lung, Colorectal and Ovarian Cancer Screening Trial. Br J Nutr. 2022;128(4):653–8. 10.1017/S0007114521003664. 10.1017/S0007114521003664 34511161

[33] Jia X, Han C (2001). Effects of green tea on colonic aberrant crypt foci and proliferative indexes in rats. Nutr Cancer.

[34] Pedro DF, Ramos AA, Lima CF, Baltazar F, Pereira-Wilson C (2016). Colon Cancer Chemoprevention by Sage Tea Drinking: Decreased DNA Damage and Cell Proliferation. Phytother Res.

[35] Lee KJ, Inoue M, Otani T, Iwasaki M, Sasazuki S, Tsugane S (2007). Coffee consumption and risk of colorectal cancer in a population-based prospective cohort of Japanese men and women. Int J Cancer.

[36] Wada K, Oba S, Tsuji M, Goto Y, Mizuta F, Koda S, Uji T, Hori A, Tanabashi S, Matsushita S (2019). Green tea intake and colorectal cancer risk in Japan: the Takayama study. Jpn J Clin Oncol.

[37] Zhang X, Albanes D, Beeson WL, van den Brandt PA, Buring JE, Flood A, Freudenheim JL, Giovannucci EL, Goldbohm RA, Jaceldo-Siegl K (2010). Risk of colon cancer and coffee, tea, and sugar-sweetened soft drink intake: pooled analysis of prospective cohort studies. J Natl Cancer Inst.

[38] Wang XJ, Zeng XT, Duan XL, Zeng HC, Shen R, Zhou P (2012). Association between green tea and colorectal cancer risk: a meta-analysis of 13 case-control studies. Asian Pac J Cancer Prev.

[39] Wang ZH, Gao QY, Fang JY (2012). Green tea and incidence of colorectal cancer: evidence from prospective cohort studies. Nutr Cancer.

[40] Yu F, Jin Z, Jiang H, Xiang C, Tang J, Li T, He J (2014). Tea consumption and the risk of five major cancers: a dose-response meta-analysis of prospective studies. BMC Cancer.

[41] Zhu MZ, Lu DM, Ouyang J, Zhou F, Huang PF, Gu BZ, Tang JW, Shen F, Li JF, Li YL (2020). Tea consumption and colorectal cancer risk: a meta-analysis of prospective cohort studies. Eur J Nutr.

[42] Kim TL, Jeong GH, Yang JW, Lee KH, Kronbichler A, van der Vliet HJ, Grosso G, Galvano F, Aune D, Kim JY (2020). Tea consumption and risk of cancer: an umbrella review and meta-analysis of observational studies. Adv Nutr.

[43] Hu Y, McIntosh GH, Le Leu RK, Somashekar R, Meng XQ, Gopalsamy G, Bambaca L, McKinnon RA, Young GP (2016). Supplementation with Brazil nuts and green tea extract regulates targeted biomarkers related to colorectal cancer risk in humans. Br J Nutr.

[44] Morris J, Moseley VR, Cabang AB, Coleman K, Wei W, Garrett-Mayer E, Wargovich MJ (2016). Reduction in promotor methylation utilizing EGCG (epigallocatechin-3-gallate) restores RXRα expression in human colon cancer cells. Oncotarget.

[45] Peluso I, Serafini M (2017). Antioxidants from black and green tea: from dietary modulation of oxidative stress to pharmacological mechanisms. Br J Pharmacol.

[46] Yuan X, Long Y, Ji Z, Gao J, Fu T, Yan M, Zhang L, Su H, Zhang W, Wen X (2018). Green Tea Liquid Consumption Alters the Human Intestinal and Oral Microbiome. Mol Nutr Food Res.

[47] Hao X, Xiao H, Ju J, Lee MJ, Lambert JD, Yang CS (2017). Green tea polyphenols inhibit colorectal tumorigenesis in azoxymethane-treated F344 rats. Nutr Cancer.

[48] Ward RE, Benninghoff AD, Healy BJ, Li M, Vagu B, Hintze KJ. Consumption of the total Western diet differentially affects the response to green tea in rodent models of chronic disease compared to the AIN93G diet. Mol Nutr Food Res. 2017;61(4). 10.1002/mnfr.201600720. 10.1002/mnfr.201600720 27921383

[49] Wang ST, Cui WQ, Pan D, Jiang M, Chang B, Sang LX (2020). Tea polyphenols and their chemopreventive and therapeutic effects on colorectal cancer. World J Gastroenterol.

